# Psychological Wellbeing, Worry, and Resilience-Based Coping during COVID-19 in Relation to Sleep Quality

**DOI:** 10.3390/ijerph19010050

**Published:** 2021-12-21

**Authors:** Olivia H. Tousignant, Sarah W. Hopkins, Abigail M. Stark, Gary D. Fireman

**Affiliations:** 1 Psychology Department, Suffolk University, Boston, MA 02108, USA; shopkins@su.suffolk.edu (S.W.H.); gfireman@suffolk.edu (G.D.F.); 2 Harvard Medical School, McLean Hospital, Belmont, MA 02478, USA; amstark@mgh.harvard.edu

**Keywords:** rumination, mindfulness, worry, coping strategies, COVID-19, sleep

## Abstract

The current study evaluated the impact of psychological wellbeing on sleep quality during the onset of the COVID-19 pandemic. A novel empirical model tested variables that mediate and moderate this impact. First, a relationship was established between psychological wellbeing during the COVID-19 pandemic and sleep quality. Second, resilience-based coping associated with the COVID-19 pandemic was tested as a mediator of the impact of psychological wellbeing on sleep quality. Third, dispositional rumination, mindfulness, and worry were compared as moderators of the impact of psychological wellbeing on sleep quality. Fourth, a moderated mediated model was tested for each moderator. Online survey data was collected from 153 adults in the United States. Results demonstrated that coping with the COVID-19 pandemic partially mediated the impact of psychological wellbeing on sleep quality. Worry, but not rumination or mindfulness, moderated the impact. A moderated mediation model failed to demonstrate significance, indicating that the data are best represented by distinct mediation and moderation models. Thus, interventions aimed at improving sleep quality should prioritize concurrent reduction in worry and increase in resilience-based coping strategies. This study provides practical and theoretical contribution to the literature by demonstrating relationships between key variables and contextualizing how the model can be used for assessments and interventions during widespread crises.

## 1. Introduction

Sleep quality is integral to wellbeing and quality of life [1,2,3,4]. It has been found to be impacted by a host of variables including stress, fear, and anxiety as well as perseverative thinking. To date, an abundance of research has examined the many influences of psychological variables on sleep quality, and a complex multifactor picture has emerged.

Recently, investigations exploring individuals’ sleep during the COVID-19 pandemic point to patterns of diminished sleep quality as well as collective disruptions to daily life [5]. Sleep quality is known to be shifted or moderated by stress [6], but the pandemic presented global changes and concerns beyond typical shifting stress levels. Thus, it is no surprise that studies have demonstrated diminished sleep quality as a result of COVID-19 related stressors and collective issues. It is important to note that although the pandemic affected individuals globally, some individuals were faced with a higher number of stressors (e.g., job loss, death of a loved one) than were other individuals and communities. In understanding the effect of COVID-19 on sleep quality [7], it is important to also assess levels of stress and eustress associated with the adjustment to the pandemic.

Research demonstrates that many individuals responded with resilience and effective coping during the pandemic, which likely has ameliorated some of the negative effects of stress on their sleep quality. For example, during the pandemic, resilience-based coping was found to mediate the impact of fatigue on clinical nurses’ sleep quality [8]. Due to the continuation of the COVID-19 pandemic and the unfortunate inevitability of future large-scale collective crises, an increased understanding of the relation between psychological wellbeing, resilience-based coping, and sleep quality is integral for effectively addressing health needs.

### 1.1. Main Effect Model: Psychological Wellbeing

In conceptualizing the current study, psychological wellbeing refers to the summative impact of the pandemic on multiple areas of people’s lives. There is currently a paucity of research examining individuals’ overall perceived psychological wellbeing during the COVID-19 pandemic. Measures of wellbeing used in prior research during the pandemic either have tended to model COVID-19-specific stress [9,10,11,12] instead of wellbeing or have only measured general wellbeing rather than a more comprehensive pandemic-related wellbeing [13,14]. Research demonstrates that restoration of a single area of life disruption, such as daily habits, does not necessarily improve perceived wellbeing [15]. As such, there remains a need to develop a more comprehensive assessment of how psychological variables operate within a pandemic. Psychological wellbeing should be examined in a more holistic manner, incorporating many areas of life when representing the broad psychological experience that predicts sleep quality.

On the basis of past research, the current authors created a novel measure of psychological wellbeing during the COVID-19 pandemic to address the current study’s research questions. The novel measure of psychological wellbeing contains 13 areas: (a) lifestyle and daily routines [16]; (b) general stress level [9]; (c) general sleep health beyond sleep quality [17]; (d) emotional health [18]; (e) physical health [19]; (f) sexual health [20]; (g) access to resources [21] such as food [22], toilet paper [23], cleaning supplies [24], heat [25], and electricity [26]; (h) job security [27]; (i) financial security [28]; (j) housing stability [29]; (k) sense of safety [30]; (l) sense of privacy [31]; and (m) sense of belonging to a social community [32]. These factors were selected because prior research has demonstrated their importance in contributing to psychological wellbeing. While individuals adjusted to changes during the COVID-19 pandemic, some areas may have been negatively changed, some may have been positively changed, and some may have remained stable. The novel measure not only examines changes in psychological wellbeing during the COVID-19 pandemic, but it also measures differential valences of wellbeing in terms of improvements or detriments. Therefore, psychological wellbeing is considered to reflect one’s cumulative degree of change.

### 1.2. Mediation Model: Resilience-Based Coping

Research demonstrates that it is not only psychological wellbeing and stress that impact sleep quality but also how one copes with stress [33]. In order for us to more fully understand how psychological wellbeing influences sleep quality during the pandemic, coping strategies specific to the COVID-19 pandemic must be considered. Past research examining the relationship between psychological wellbeing, coping, and sleep quality has neither examined these same three variables within one model nor within the context of the COVID-19 pandemic.

Resilience-based coping has been identified as a mediator of the relationship between negative life events and psychological wellbeing [34]; however, it is unclear as to how resilience-based coping relates to sleep quality. Further, the relationship between resilience-based coping and sleep quality during a global crisis, specifically, has not been elucidated to date. Therefore, the current study examines how commonly used resilience-based coping strategies in response to the COVID-19 pandemic indirectly affect the impact of perceived psychological wellbeing on sleep quality.

### 1.3. Moderation Model: Cognitive Approaches

When a wider lens is used to examine the model of study it is to explore how one’s disposition or cognitive approach augments the impact of psychological wellbeing on sleep quality. Cognitive approaches involve the nature of one’s mentation process during stressful situations. Importantly, the cognitive approaches assessed in the current study were not asked in direct relation to the COVID-19 pandemic. Thus, they were conceptualized as possible moderators that could impact how strongly changes due to the COVID-19 pandemic disrupted an individual’s life experience (psychological wellbeing) and ability to cope with this stress (resilience-based coping). The three cognitive approaches are considered to be dispositional attributes that could strengthen or weaken one’s perceived wellbeing and coping to impact sleep quality.

The current study explores the impact of three types of cognitive approaches: dispositional rumination (perseverative negative thoughts about the past), dispositional mindfulness (nonjudgmental thoughts about the present), and dispositional worry (perseverative negative thoughts about the future). These are three important cognitive approaches that may moderate connections between psychological wellbeing, resilience-based coping, and sleep quality. Research suggests that the experience of both rumination and worry magnify negative life components and minimize positive areas of life [35,36]. Such maladaptive negative focus is associated with reduced wellbeing [36,37]. In contrast, the tendency to approach life with a more present-focused cognitive set, by mindfully experiencing life’s many changing occurrences, is associated with greater wellbeing [38,39].

To date, there is limited consensus about the relative degrees to which rumination, worry, and/or mindfulness differentially augment the relationship between psychological wellbeing and sleep quality. Information gleaned from the comparison of dispositional rumination, mindfulness, and worry as moderators can inform rationale for prioritizing certain interventions and health promotion efforts during stressful life events and crises.

Overall, there remains a need for clarity in how these constructs—psychological wellbeing; resilience-based coping; and the cognitive approaches of rumination, worry, and mindfulness—operate in relation to each other to influence sleep quality. After the main effects, mediation, and moderations are compared, the final study aim is to examine whether a combined moderated mediation model exists between these variables when impacting sleep quality. A moderated mediation model could elucidate whether to consider these constructs as synergistically operating versus independently operating.

The study hypotheses:

**Hypothesis** **1** **(H1).** *Psychological wellbeing during the COVID-19 pandemic will predict sleep quality.*

**Figure 1:**
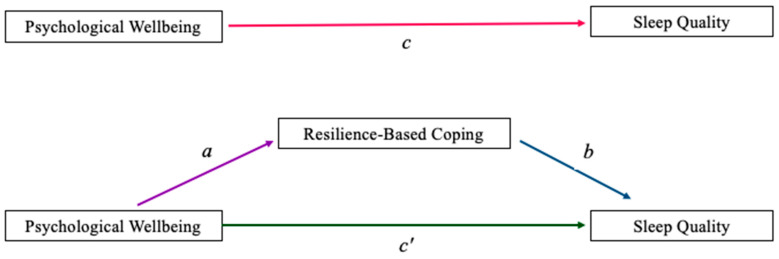
Top panel depicts the potential direct effect of psychological wellbeing on sleep quality. Bottom panel depicts the potential mediation pathway through which resilience-based coping may impact sleep quality.

**Hypothesis** **2** **(H2).** *A statistically significant relationship between psychological wellbeing during the pandemic and sleep quality will remain when accounting for the potential mediating effect of resilience-based coping with the COVID-19 pandemic (Figure 1).*

**Figure 2:**
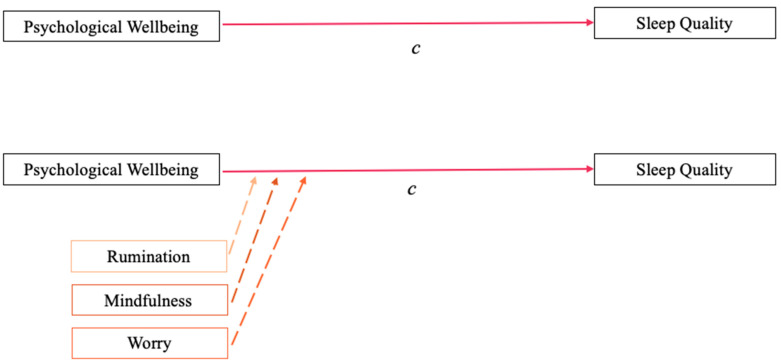
Moderation depicting that rumination, mindfulness, and worry each may augment the impact of psychological wellbeing on sleep quality.

**Hypothesis** **3** **(H3).** *Dispositional rumination, worry, and mindfulness each will moderate the impact of psychological wellbeing during the COVID-19 pandemic on sleep quality (Figure 2).*

**Figure 3:**
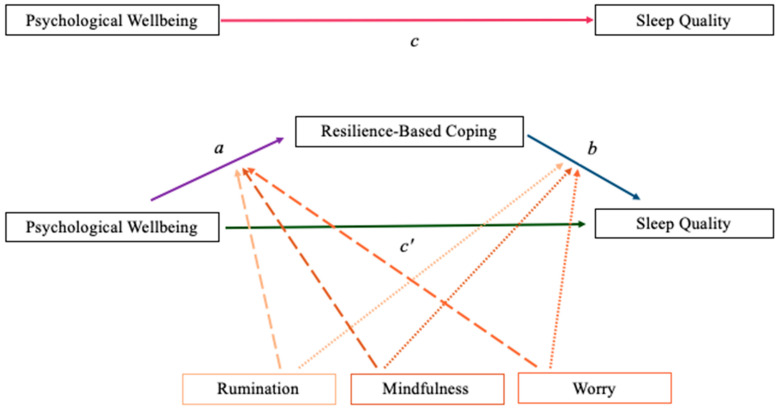
Moderated mediation depicting potential pathways through which rumination, mindfulness, and worry may impact sleep quality.

Additionally, the study addresses the following question:

**Hypothesis** **4** **(H4).** *When Hypotheses 2 and 3 are analyzed, do the variables operate as two distinct models (moderation and mediation) or as a combined moderated mediation (Figure 3)?*

## 2. Materials and Methods

### 2.1. Procedures and Participants

The current study methods were approved by the Institutional Review Board (IRB # 1455469-3). There were no conflicts of interest to disclose. The Mechanical Turk (MTurk) and Qualtrics survey platforms were used to collect data from 153 adult participants in the United States general population. Data was collected in May 2020 and June 2020 during the initial phases of the COVID-19 pandemic. Eligibility criteria required that participants were at least 18 years of age, fluent in English, had a minimum of a high school degree from the United States, and had regular access to the Internet. Exclusion criteria required that participants were not taking sleep medications or medications that depress or activate the central nervous system, were not addicted to alcohol or drugs, and did not work overnight shifts. For quality data control, only participants with a history of satisfactory approval ratings and feedback on previous tasks (95% approval rating for previous MTurk tasks completed) were allowed to access the study. Participants were compensated USD 7 for participating in the study, commensurate with research on MTurk participation [40]. For approximately 20 min, participants completed surveys about their sleep experiences, their emotional experiences, and their experiences adjusting to and resiliently coping with the pandemic. Demographics of the sample are described in Table 1.

### 2.2. Measures

Pittsburgh Sleep Quality Index (PSQI) [41]: This sleep quality Likert scale assessed participants’ subjective global appraisal of their sleep quality over the past month. The item that directly assesses participants’ sleep quality, *During the past month, how would you rate your sleep quality overall?*, was used to ensure most direct measurement of participants’ appraisal of their quality of sleep. Participants select from the four options, reverse scored as: *1 = very bad, 2 = fairly bad, 3 = fairly good, 4 = very good*. The one-item measure was chosen to focus on how sleep quality, specifically, is predicted by psychological wellbeing. There is precedent for a one-item measure of subjective sleep quality used in prior research [42,43,44]. Participants’ average level of sleep quality in the past month was 2.84 (*SD* = 0.78, *range* = 1–4). The sleep quality variable was normally distributed with no excess kurtosis.

**Figure 4:**
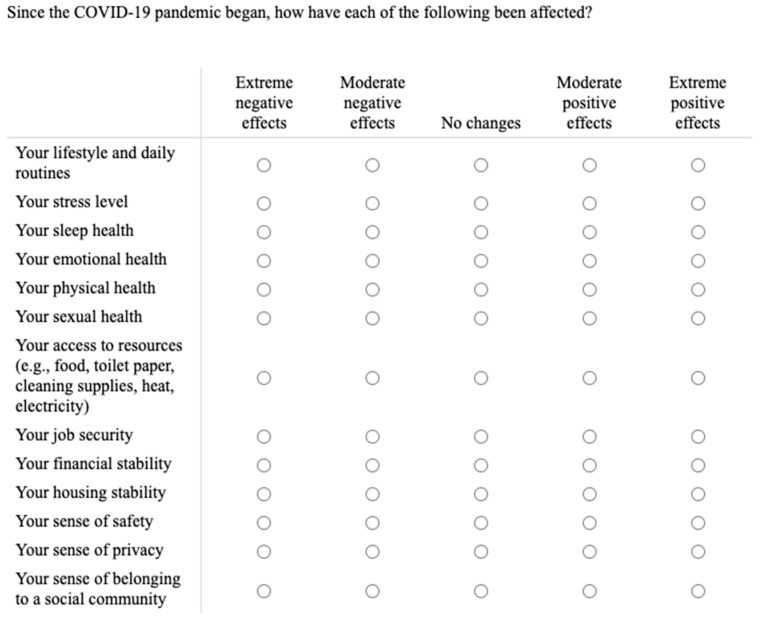
The Effects of Abiotic and Biotic Crises (EBAC-13) is a measure of perceived psychological wellbeing while adjusting to a community wide crisis.

Effects of Biotic and Abiotic Crises—13 Item (EBAC-13; Figure 4): This scale was designed [45] to account for the variety of impacts that the COVID-19 pandemic could have on health and wellbeing. The construction of the scale was based on prior research examining human impacts stemming from natural disasters including the 9/11 terrorist attacks and the Ebola virus outbreak. While other general wellbeing measures exist [13,46,47], none to date have been published to directly assess overall psychological wellbeing as related to one’s adjustment to the pandemic. The matrix design of the measure (Figure 4) allows ease of use and reduced burden on participants. Additionally, to increase flexibility for use of the measure in future research, one can substitute the sentence stem, “Since the COVID-19 pandemic began,…” with the names of other widespread crises that occur in the future as, “Since the [add event name here] began,…” to aid understanding of individuals’ perceived adjustment.

Ratings are on a 5-point Likert scale with higher scores indicating greater psychological wellbeing. A cumulative score of the 13 items is calculated such that *extreme negative effects = 1, moderate negative effects = 2, no changes = 3, moderate positive effects = 4,* and *extreme positive effects = 5.* Therefore, the possible range of total scores is 13–65, with higher cumulative scores reflecting greater psychological wellbeing or positive impacts. Participants’ average level of psychological wellbeing during the adjustment to the COVID-19 pandemic was 36.2 (*SD* = 7.79, *range* = 20–62). Psychological wellbeing was slightly positively skewed, as would be expected while adjusting to the onset of the pandemic. *Cronbach’s α* for the *EBAC-13* was 0.904, indicating high reliability or inter-item consistency of the measure. Bartlett’s Test of Sphericity demonstrated rejection of a null identity matrix, showing a multidimensional unifactorial model of psychological health, *X*^2^ (1, *n* = 153) = 885.98, *df* = 78, *p* < 0.001. Rejection of the null implies that the space between diagonal and off-diagonal elements is not uniformly distributed but rather is clustered.

Resilience-Based Coping: The Brief Resilient Coping Scale [48] is a four-item measure. It has robust empirical precedence, having been used to assess general abilities to cope with stress adaptively and proactively within a resilience framework. Participants rate the degree to which they agree that they use each of four types of coping strategies. Ratings are made on a five-point Likert scale: *1 = does not describe me at all; 5 = describes me very well.* Total scores range from 4 to 20, with higher scores indicating higher resilience-based coping. Participants’ average level of psychological wellbeing while adjusting to the COVID-19 pandemic was 36.2 (*SD* = 7.79, *range* = 20–62). The resilience-based coping variable was negatively skewed demonstrating that the modal number of participants had high coping scores, as would be expected in the general population. The Brief Resilient Coping Scale has been found to have good criterion validity with empirically validated measures of mental health, optimism, self-efficacy, self-esteem, and wellbeing [49]. For the current study, the sentence stem was changed from “Generally in response to stress,…” to “In response to COVID-19 pandemic stress,…”, yet both forms of the scale were included in the method to confirm convergent validity. There was strong convergent validity between the general scale and the COVID-19 scale (*r* = 0.74, *p* < 0.001). The average level of resilience-based coping for the participants during the pandemic was 14.9 (*SD* = 3.2, *range* = 4–20) and the scale had moderate internal reliability (*Cronbach’s α* = 0.785).

Rumination Response Styles to Depression Questionnaire (RSDQ) [50,51]: This questionnaire measures an individual’s general tendency to ruminate about the past. Participants complete 22 items assessing the tendency to engage in perseverative negative thinking about the past as a cognitive response style. Participants rate each item on a four-point Likert scale wherein *1 = almost never, 2 = sometimes, 3 = often,* and *4 = almost always.* A summative score is calculated with a possible range of 22 to 88, with higher scores reflecting a higher level of rumination. Within our sample, this measure had high internal reliability (*Cronbach’s α* = 0.964). The average rumination score was 41.45 (*SD* = 15.2, *range* = 22–81).

Five Facet Mindfulness Questionnaire—15 item (FFMQ-15) [38,52]: This questionnaire assesses an individual’s general tendency towards mindfulness in daily life. Participants complete 15 items measuring five facets of mindfulness: observing, acting with awareness, nonjudging, describing, and nonreactivity [53]. Participants rate each item on a five-point Likert scale wherein *1 = never or very rarely true; 5 = very often or always true*. Total scores range from 15 to 75, with higher cumulative scores indicating greater tendency to be mindful in daily life. Factor analyses have provided support for calculating a summative score to reflect overall trait mindfulness [54]. Thus, in the current study a summative score for each participant was calculated. Within our sample, the FFMQ-15 had moderate internal reliability (*Cronbach’s α* = 0.714) and the average mindfulness score was 51.8 (*SD* = 7.9, *range* = 30–73).

Penn State Worry Questionnaire (PSWQ) [55]: This questionnaire measures an individual’s general tendency to worry about the future. Participants use a five-point Likert scale wherein *1 = not at all typical of me; 5 = very typical of me* to complete 16 items assessing the tendency to engage in perseverative negative thinking about the future as a cognitive response style. A summative score is calculated with a possible range of 16 to 80. Higher scores reflect greater tendency to worry, accounting for frequency, global, and uncontrollable dimensions. Within our sample, this measure had high internal reliability (*Cronbach’s α* = 0.955) and the average worry score was 46.94 (*SD* = 17.0, *range* = 16–80).

### 2.3. Data Analysis

All analyses were conducted using SPSS version 27. An a priori power analysis was conducted using G*Power software to determine the minimum sample size necessary for the current study [56,57]. The power analysis was based on an alpha probability level of 0.05, a statistical power level of 0.95, and an anticipated effect size of 0.80. Each of the 153 participants included in the analyses had complete data for each of the six measures. Levels of skewness were in acceptable ranges. Correlational analyses indicated that the six constructs assessed were related yet independent constructs. Psychological wellbeing while adjusting to the pandemic was positively associated with resilience-based coping (*r* = 0.27, *p* < 0.001), sleep quality (*r* = 0.22, *p* = 0.006), and rumination (*r* = 0.16, *p* = 0.045); psychological wellbeing was inversely associated with worry (*r* = −0.20, *p* = 0.013). Psychological wellbeing was not associated with mindfulness. Resilience-based coping was positively associated with sleep quality (*r* = 0.31, *p* < 0.001) and mindfulness (*r* = 0.38, *p* < 0.001); resilience-based coping was inversely associated with rumination (*r* = −0.22, *p* = 0.007) and worry (*r* = −0.34, *p* < 0.001). Sleep quality in the past month was positively associated with mindfulness (*r* = 0.37, *p* < 0.001); sleep quality was inversely associated with rumination (*r* = −0.32, *p* < 0.001) and worry (*r* = −0.49, *p* < 0.001).

Mediation analyses were conducted using methods described by Baron and Kenny (1986) [58] in combination with the Monte Carlo website for calculating confidence intervals [59]. Moderation analyses were conducted by mean-centering all variables and creating interaction terms, which were entered into linear regressions testing psychological augmentation of how COVID-19-related psychological wellbeing predicts sleep quality. Given the conceptual distinction between situational resilience-based coping versus dispositional cognitive approaches when analyzing Hypotheses 2 and 3, respectively, we developed an empirical question in order to examine whether moderated mediations exist. Examining the linked nature of the six psychological variables of interest—psychological wellbeing (x), resilience-based coping (mediator), sleep quality (y), rumination (moderator 1), mindfulness (moderator 2), and worry (moderator 3)—the current authors tested the moderated mediation models (Figure 3).

## 3. Results

### 3.1. Main Effect Model Analysis and Mediation Model Analysis

Examining the first hypothesis through regression (Table 2), we found a statistically significant impact of psychological wellbeing (EBAC) on sleep quality (SQ). A statistically significant main effect was observed, demonstrating that sleep quality was impacted by psychological wellbeing during the adjustment to the COVID-19 pandemic (*c-path*; *B* = 0.02, *t* = 2.77, *p* = 0.006, *β* = 0.221). Regression analyses were conducted to test the second hypothesis that the impact of EBAC on SQ is mediated or indirectly effected by resilience-based coping with COVID-19 (RCC). Results indicated that EBAC was a statistically significant predictor of RCC (*a-path*; *B* = 0.11, *t* = 3.47, *p* < 0.001, *β* = 0.27) and that RCC was a statistically significant predictor of SQ (*b-path*; *B* = 0.07, *t* = 4.02, *p* < 0.001, *β* = 0.31). The *ab-path* was also statistically significant. Percent mediation indicated that RCC accounts for approximately 33.52% of the relationship between EBAC-13 and SQ. When controlling for RCC, we found that the statistically significant main effect of EBAC on SQ remained (*c’-path*; *B* = 0.02, *t* = 2.19, *p* = 0.030, *β* = 0.17). These results provide support for resilience-based coping as a partial mediator of the relationship between psychological wellbeing and sleep quality.

### 3.2. Analyses of Cognitive Approach Moderating the Impact of Psychological Wellbeing on SQ

**Figure 5:**
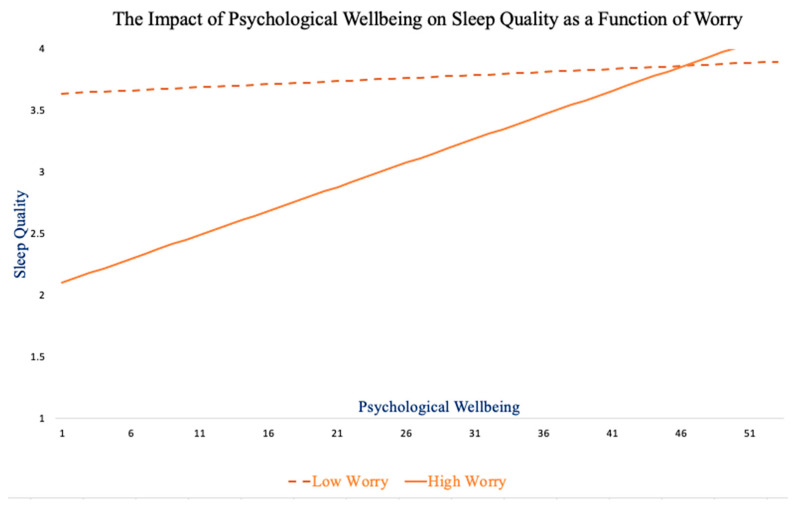
The effect of psychological wellbeing on sleep quality changes across worry levels. The point at which the two lines intersect represents when the impact of psychological wellbeing on sleep quality is equivalent across participants.

Three hierarchical multiple regression analyses were conducted to examine rumination (RUM), worry (WOR), and mindfulness (MNDFL) as potential moderators of the relationship between EBAC and SQ (Table 3). In step 1 of the analyses, the predictor and moderator variables (mean-centered) were added to assess the main effect of EBAC on SQ. In step 2, an interaction term computed by multiplying the two mean-centered predictors (i.e., RUM*EBAC, MNDFL*EBAC, WOR*EBAC) was added. Results indicated that the interaction of worry and psychological wellbeing (WOR*EBAC) accounted for a statistically significant amount of the variance in SQ above and beyond the main effect of EBAC on SQ (*ΔR*^2^ = 0.03, *p* = 0.028). Moderation effects were neither found for rumination nor for mindfulness. Figure 5 depicts the nature of the worry driven interaction, demonstrating that the impact of EBAC on SQ changes across levels of worry. For low worriers (one standard deviation below the mean), SQ stayed relatively stable, regardless of whether low worriers experienced low or high EBAC. High worriers (one standard deviation above the mean) had lower SQ, as expected, and the quality of their sleep improved as their EBAC increased. These results indicate that worry, specifically, is a dispositional moderator of the impact of psychological wellbeing on sleep quality.

### 3.3. Moderated Mediation Model Analyses

To determine if the variables operate in tandem through moderated mediation, beyond a distinct mediation and a distinct moderation model, we used the Hayes Process v4.0 extension in SPSS. Prior research has indicated that a minimum of 80 participants—20 participants per variable within the model—is needed to test for moderated mediation [60]. When the three moderated mediation analyses were tested as three separate models (Figure 3), none yielded statistically significant results. Rumination did not moderate the mediation (*CI* = −0.0003, 0.0002), mindfulness did not moderate the mediation (*CI* = −0.0005, 0.0005), and worry did not moderate the mediation (*CI* = −0.0003, 0.0002). The main effect model remained stable (Figure 1, panel a), the mediation model endured (Figure 1, panel b), and the moderation model of worry stayed intact (Figure 2, worry; Figure 5).

## 4. Discussion

The broad impact of the COVID-19 pandemic on communities and individuals fundamentally changed daily life, learning, work, and play. Prior psychological research on the pandemic has primarily focused on pandemic related stress or on specific emotional, behavioral, and environmental concerns. Depending on their contexts, individuals throughout the world have had different experiences during the pandemic and their sleep quality has been affected in different ways. The current study demonstrates that people with better psychological wellbeing had better sleep quality, whereas people with poorer psychological wellbeing had poorer sleep quality. To our knowledge, this is the first study to demonstrate this link in the context of the pandemic.

### 4.1. Main Effect Model and Mediation Model

Supporting our first hypothesis, a statistically significant association was found between psychological wellbeing and sleep quality during the pandemic (Figure 1, panel a). Supporting our second hypothesis, resilience-based coping significantly mediated the impact of psychological wellbeing on sleep quality (Figure 1, panel b).

Approximately 33.52% of the impact of psychological wellbeing on sleep quality was accounted for by resilience-based coping. Increased psychological wellbeing was associated with higher levels of resilience-based coping, which was associated with better sleep quality. Conversely, decreased psychological wellbeing was associated with lower levels of resilience-based coping, which was associated with poorer sleep quality. Such relational directionality suggests that if someone with relatively low psychological wellbeing proactively engages in resilience-based coping, their sleep quality is better supported. These results are in line with other recent studies on sleep quality during the pandemic, which have shown that change in sleep quality varies based on individual differences [7,61,62]; however, the current study is the first to examine individual differences in situational coping as impacting sleep quality during the pandemic. 

Notably, there was a partial rather than a full mediation, indicating that the main effect of psychological wellbeing on sleep quality is an independently valuable path to continue investigating. Regardless of one’s proactive engagement in resilience-based coping, higher psychological wellbeing predicted better sleep quality while adjusting to the pandemic. These findings substantially advance the literature by demonstrating a strong relationship between psychological wellbeing and sleep quality [63]. Prior to this study, relationships among these variables had primarily been in clinical samples and other more narrowly defined samples, such as individuals with diabetes [1] and nurses or healthcare worker samples [2]. The current study examined these relationships in a general population of adults in the United States, extending the model’s applicability and generalizability. Future research demonstrating how these constructs operate in other cultures will be instrumental for detecting and addressing sleep quality vulnerabilities on a global scale.

When health research and public health policy are considered, it is recommended that increased efforts are made to disseminate information about resilience-based coping strategies [64]. The strategies examined were (1) looking for creative ways to alter difficult situations, (2) believing that one has control over their reaction to it, (3) believing that one can grow in positive ways by dealing with difficult situations, and (4) actively seeking ways to replace the losses one encounters in life. Importantly, the same patterns of mediation emerged when the strategies were combined to create a total score as when they were examined separately. This suggests that each of the resilience-based coping strategies is similarly influential on sleep quality. Therefore, when aiming to improve their coping and sleep quality during the pandemic, individuals may practice the strategy that is most suitable in terms of context and personal preferences.

### 4.2. Moderation Model

In support of the hypothesis that dispositional cognitive approach would moderate the impact of psychological wellbeing on sleep quality, dispositional worry was found to have a statistically significant effect. Consistent with prior research, the current study found evidence of a relationship between psychological wellbeing, worry, and sleep quality [65,66]. The interacting elements of psychological wellbeing and worry have a compounding impact on sleep quality. These findings advance the literature by demonstrating that worry, but not rumination or mindfulness, augments the impact of psychological wellbeing on sleep quality.

Prior theory has suggested that worry and rumination operate in a similar manner to impact the effect of stress on sleep quality, with research indicating comparable magnitudes of effect for worry and rumination as moderators of the association between stress and sleep quality [44]. Worry and rumination are often considered overlapping concepts in that they both involve perseverative negatively valenced thinking [67]. Advancing existing theory, the current results suggest that the future-oriented aspect of worry is an especially salient variable that impacts sleep quality during the pandemic. Accordingly, people who are high worriers are particularly susceptible to their sleep quality being impacted by their level of psychological wellbeing. This pronounced impact of worry is consistent with literature indicating that increased levels of COVID-19-related worry were associated with poor sleep quality in young adults [68].

Finally, addressing the study’s exploratory fourth hypothesis regarding a moderated mediation model, the current study results demonstrate that a moderation model and mediation model exist distinctly or independently rather than a synergistically. When moderated mediation analyses were tested for the three cognitive approaches, none were statistically significant. This indicates that the dispositional cognitive approach does not interact with the effect of the mediator; rather, separate mediation and moderation processes are operating.

### 4.3. Future Directions and Limitations

This study has several limitations to be addressed in future research. Neither rumination nor mindfulness was found to moderate the impact of psychological wellbeing on sleep quality. This is inconsistent with prior studies that have found a relationship between rumination and specific domains of wellbeing (e.g., social support) in influencing sleep quality [69]. The question emerges as to whether psychological wellbeing alters the connection between rumination and mindfulness with current wellbeing and sleep during a pandemic [67].

Additionally, this study used a sample of predominately White adults who had graduated from high school in the United States. This represents a clear limitation in the generalizability of the results outside of this population. Given the global effects of the pandemic [70], further research is needed to better understand connections between wellbeing, coping, sleep, and cognitive dispositions in a variety of populations. With the widespread impacts of the pandemic and other disasters, this model should be tested in other cultures outside of the United States. More research will clarify how coping strategies and cognitive dispositions operate to impact sleep quality depending on the context of the population.

Future research should focus on better understanding the processes involved in these relationships. As the current study found separate mediation and moderation models, assessing whether this continues to be the case in replication studies is warranted. Longitudinal analysis of the EBAC-13 measure within the context of these models would help advance knowledge about change over time in the relationship between psychological wellbeing and sleep quality. For example, Kocevska and colleagues (2020) found an association between variability in sleep quality and levels of worry. Additionally, for about a quarter of their participants, Kocevska and colleagues (2020) found a paradoxical effect of the pandemic on sleep quality: people with poor pre-pandemic sleep experienced better sleep during the pandemic, whereas people with good pre-pandemic sleep experienced poorer sleep during the pandemic. These results indicate that individual differences are essential to study longitudinally to build knowledge about the impact of psychological variables on sleep quality. Worry and psychological wellbeing seem to be two particularly influential variables for understanding sleep quality during the pandemic.

In addition, resilience-based coping was found to account for a notable portion of the variance in the impact of psychological wellbeing on sleep quality. All together, the current study findings are important for research on public health and wellbeing. They suggest that interventions may be more effective when designed to concurrently target *both* the process of worrying *and* how one engages in resilience-based coping. The current study findings suggest that, to support good sleep quality during the COVID-19 pandemic, individuals can (a) reduce engagement in future-oriented negative thought and (b) increase engagement in proactive coping. Findings suggest that these methods have the greatest impact for individuals reporting low levels of psychological wellbeing.

Dismantling research should be conducted to examine more precisely whether certain elements of coping strategies and cognitive dispositions are more relevant than others for influencing sleep quality when adjusting to crises. One such variable that may be operating is the element of control [6,71,72]. Controlling one’s constructive response to situational stress can be considered a form of proactive resilience-based coping [73]. Future studies should examine psychological control and other potential mechanisms to better understand the underlying nature of how the key variables interact. Finally, it would be empirically valuable to examine how these factors operate in those with identified risk factors such as in clinical populations.

## 5. Conclusions

In sum, the current study demonstrates that psychological wellbeing independently predicts sleep quality when adjusting to the pandemic. Resilience-based coping partially mediates this relationship. Worry, specifically, moderates the effect of psychological wellbeing on sleep quality, whereas mindfulness and rumination do not exert moderating influence. Taken together, these results extend the literature by differentiating the degrees to which sleep quality is influenced by psychological variables during the pandemic. The current study highlights that the psychological impact of the pandemic is best understood within a larger framework integrating coping and cognitive approaches. Replication studies should be conducted across cultures as well as during unfortunately inevitable future collective crises. Overall, the current study results demonstrate the nature of multifaceted relationships between psychological wellbeing, situational resilience-based coping, dispositional cognitive approaches, and sleep quality during the COVID-19 pandemic.

## Figures and Tables

**Table 1:** *n* = 153. Participants ranged in age from 20 to 72 years, and the average age was 43.23 years (*SD* = 12.86).

*Demographic* *Characteristic*	*n*	%
Gender		
Female	76	49.7
Male	76	49.7
Non-binary	1	0.6
Race		
White	125	81.7%
Asian	9	5.9%
Black	6	3.9%
Multiracial	13	8.5%

**Table 2:** Coefficients showing the paths of the mediation analysis (Figure 1).

	Unstandardized Coefficient	Standardized Coefficient		
Effect	*B*	*Beta*	*t*	*p*
*c-path:* Wellbeing → Sleep quality	0.02	0.221	2.77	0.006
*a-path:* Wellbeing → Resilience-based coping	0.11	0.27	3.47	<0.001
*b-path:* Resilience-based coping → Sleep quality	0.07	0.31	4.02	<0.001
*c’-path:* Wellbeing → Resilience-based coping → Sleep quality	0.02	0.17	2.19	0.030

**Table 3:** *n* = 153. EBAC = The Effects of Biotic and Abiotic Crises—13 items (EBAC-13) measuring psychological wellbeing. RUM = rumination, MINDFL = mindfulness, and WOR = worry. CI = confidence interval; LL = lower limit; UL = upper limit. The table below represents the three moderations that were tested.

	Size of Effect	95% CI		
	*R^2^*	*LL*	*UL*	*p*
Step 1 - Rumination Model				
Constant		2.696	2.948	<0.001
Step 2 - Rumination Interaction				
RUM*EBAC	1.8%	0.000	0.002	0.104
Step 1 – Mindfulness Model				
Constant		2.710	2.962	<0.001
Step 2 – Mindfulness Interaction				
MNDFL*EBAC	0.4%	-0.003	0.001	0.426
Step 1 – Worry Model				
Constant		2.742	2.998	<0.001
Step 2 – Worry Interaction				
WOR*EBAC	3.2%	0.000	0.002	0.028

## Data Availability

Data may be made available upon request to the corresponding author.
